# An evolution of socioeconomic related inequality in teenage pregnancy and childbearing in Malawi

**DOI:** 10.1371/journal.pone.0225374

**Published:** 2019-11-20

**Authors:** Gowokani Chijere Chirwa, Jacob Mazalale, Gloria Likupe, Dominic Nkhoma, Levison Chiwaula, Jesman Chintsanya

**Affiliations:** 1 Department of Economics, University of Malawi, Chancellor College, Zomba, Malawi; 2 Health Nursing and Midwifery, University of Hull, Hull, United Kingdom; 3 Health Policy Unit, University of Malawi, College of Medicine, Lilongwe, Malawi; 4 Department of Population Studies, University of Malawi, Chancellor College, Zomba, Malawi; University of Cape Coast, GHANA

## Abstract

**Background:**

Teenage pregnancies and childbearing are important health concerns in low-and middle-income countries (LMICs) including Malawi. Addressing these challenges requires, among other things, an understanding of the socioeconomic determinants of and contributors to the inequalities relating to these outcomes. This study investigated the trends of the inequalities and decomposed the underlying key socioeconomic factors which accounted for the inequalities in teenage pregnancy and childbearing in Malawi.

**Methods:**

The study used the 2004, 2010 and 2015–16 series of nationally representative Malawi Demographic Health Survey covering 12,719 women. We used concentration curves to examine the existence of inequalities, and then quantified the extent of inequalities in teenage pregnancies and childbearing using the Erreygers concentration index. Finally, we decomposed concentration index to find out the contribution of the determinants to socioeconomic inequality in teenage pregnancy and childbearing.

**Results:**

The teenage pregnancy and childbearing rate averaged 29% (*p*<0.01) between 2004 and 2015–16. Trends showed a “u-shape” in teenage pregnancy and childbearing rates, albeit a small one (34.1%; *p*<0.01) in 2004: (25.6%; *p*<0.01) in 2010, and (29%; *p*<0.01) in 2016. The calculated concentration indices -0.207 (*p*<0.01) in 2004, -0.133 (*p*<0.01) in 2010, and -0.217 (*p*<0.01) in 2015–16 indicated that inequality in teenage pregnancy and childbearing worsened to the disadvantage of the poor in the country. Additionally, the decomposition exercise suggested that the primary drivers to inequality in teenage pregnancy and child bearing were, early sexual debut (15.5%), being married (50%), and wealth status (13.8%).

**Conclusion:**

The findings suggest that there is a need for sustained investment in the education of young women concerning the disadvantages of early sexual debut and early marriages, and in addressing the wealth inequalities in order to reduce the incidences of teenage pregnancies and childbearing.

## Introduction

Teenage pregnancy and childbearing are some of the issues that most nations are endeavouring to manage in order to attain better child and maternal health outcomes by mitigating the associated challenges. Various problems arise as a result of teenage pregnancy and childbearing. Associated problems include eclampsia, which is more common among pregnant adolescent girls than among older women [1]. Additionally, complications arising from teenage pregnancy and childbirth are one of the leading causes of death globally among adolescents aged 15–19 [2,3]. Evidence also suggests that children of teenage mothers suffer more from childhood malnutrition and tend to attain low levels of education [4]. These children may also suffer from low birth weight, being born prematurely, respiratory infections, birth trauma, and perinatal mortality [4,5].

An estimated 21 million girls aged 15 to 19 years and 2 million girls aged under 15 years become pregnant every year in low-and middle-income countries (LMICs) [6]. Geographically, the sub-Saharan African region has the highest number of teenage mothers and pregnancy cases [7,8]. To address the negative outcomes of teenage pregnancy and childbearing within the framework of Universal Health Coverage (UHC), the third goal of the Sustainable Development Goals (SDGs) includes adolescent reproductive health. This is one of the key areas requiring global and national level attention and investment.

The teenage pregnancy and childbearing situation in Malawi provide a compelling case to study. First, although teenage pregnancy has declined over the decade globally, Malawi has one of the highest rates [9–11] currently at 29% of the population. Secondly, although various programmes have been implemented to ensure that girls prevent early pregnancy, especially among the poor, over the decade[12,13], the impact of such programmes provides weak evidence of the determinants of teenage pregnancy, which undermines effectiveness, and thus leads to mixed outcomes in terms of teenage pregnancies and childbearing. The economic situation in Malawi may also provide an environment for various inequality.

Malawi is located in South-East Africa and has a population of about 17.5 million [14]. The economy is largely dependent on agriculture, which accounts for almost 29.5% of the gross domestic product (GDP). Recent figures indicate that the GDP is around 6.30 billion USD and the per capita GDP is about 338.48USD [15]. With such a vast population for a country of this size, Malawi has a huge poverty problem. As of 2019, almost 51.5% of the population lives below the poverty line (the poverty line is 137,425 Kwachas per person per year) and 20.1% live in ultra-poverty. The poverty rate shows an insignificant increase in poverty levels from about 50.7% in 2010 (the poverty line was 37,002 Kwachas per person per year). However, it is a slight decline from 52.4% in the year 2004 [16,17]. The high level of poverty may also be said to be associated with increasing inequality in consumption and wealth.

Currently, the consumption Gini index of 0.450 suggests that consumption is highly pro-rich. In fact, the richest 10% of the population account for 53% of the total consumption [18]. Despite the case of consumption inequality, the wealth inequality is much higher (Gini = 0.564) [18]. The wealth inequality indicates that durable assets (radios, televisions, furniture, sewing machines, fridges, washing machines bicycles, motorcycles, and cars), are mainly owned by the rich. The high inequality and poverty rates are also reflected in the low human development index (HDI) value of 0.477. As of 2018, based on HDI, Malawi ranked 171 out of 189 countries in the world. However, this is an improvement (40.2%) from 0.340 in 1990 [19].

Although literature is replete with studies analysing various aspects of teenage pregnancy and childbearing, the focus of existing studies has been on understanding the determinants of teenage pregnancy. The factors which have been identified as some of the major determinants include early sexual activity and marriage, low educational levels, low socioeconomic status, and a lack of knowledge of reproductive health [20,21]. In addition to this, personal dispositions and habits [22] have been cited as potential confounders. Unmet needs for contraceptive use, low contraceptive use, intermittent use of contraceptives [23], family disruption, community female poverty and unemployment [24,25] are additional factors determining high levels of teenage pregnancies and childbearing in addition to a lack of power to negotiate for safer sex [26]. Recently, it has been shown that a lack of comprehensive sex education and a fear of the side effects of contraceptives are also major contributing factors [7,26,27].

To the best of our knowledge, the closest study to our paper undertakes a decomposition analysis of the socioeconomic factors associated with unintended pregnancies in Iran. The Iran study found that wealth contributes about 27% to the inequality in unwanted pregnancies [28]. Our study focuses on a different angle from those of the previously mentioned studies. While research on the determinants of teenage pregnancy has been on the rise internationally, little attention has been given in the literature to assessing the disparities in teenage pregnancy and childbearing among socioeconomic classes in developing countries, especially Malawi, and decomposition of factors that account for the disparities. Understanding the core drivers of the observed inequality in teenage pregnancy and childbearing is essential in designing effective policies that can not only improve the well-being of teenage girls but also child and maternal health in general. Furthermore, this will assist in the appropriate monitoring and evaluation of Malawi’s progress towards the achievement of the SDGs 3.1 and 3.2 of the 2030 development agenda [29].

This paper contributes to the existing literature by examining the contributors of socioeconomic-related inequality in teenage pregnancy and childbearing. Specifically, we construct concentration curves and concentration indices to explore the existence of, and measure the inequality in, teenage pregnancy and childbearing. We then undertake a trend analysis to understand the socioeconomic-related inequality in teenage pregnancy and childbearing. We finally use the decomposition analysis proposed by Erreygers (2009) to estimate the contribution of each of the determinants to the socioeconomic inequality in teenage pregnancy and childbearing.

## Materials and methods

### Data and sample

We used three rounds of the Malawi Demographic Health Survey (MDHS) of 2004, 2010 and 2015–16. MDHS is a form of a repeated cross-section study which was done by the MEASURE DHS in conjunction with the Ministry of Health and the National Statistical Office. MDHS data is available in a public repository [30]. The MDHS collected information on fertility, family planning, infant and child health and mortality, maternal health and maternal and adult mortality, child and adult nutrition, malaria, HIV and AIDS, domestic violence, orphans and vulnerable children. The respective surveys had a response rate of between 96% to 99%.

The multi-stage in the MDHS selection implies that the respondents had an equal chance of being selected. To account for the survey design, the analysis took into consideration the clustering and stratification of the survey design. While the MDHS studies collected data from reproductively active women aged 15–49, for the purposes of this paper, we used a sub-samples of adolescent girls aged between 15 and 19. Based on the survey data, there were 2,407, 5,039 and 5,273 eligible respondents in the 2004, 2010 and 2015–16 MDHS surveys, respectively giving a total of 12,719 respondents.

### Dependent variable

Our dependent variable is teenage pregnancy and childbearing. The dependent variable is a dummy which takes a value of 1 if the teenager aged between 15 and 19 in the sample had a pregnancy or had a child over the time of the study, or 0 otherwise. This is a conventional definition which is available in the MDHS and all the DHS surveys world-wide.

### Explanatory variables

We followed existing studies that had previously assessed the determinants of teenage pregnancy and, or childbearing in various countries [7,20–22,24,26,27,31,32]. Since the MDHS did not have information relating to household expenditure, which we could have used to rank households according to economic status, we therefore constructed a wealth index and used it as a measure of socioeconomic status. Construction of the wealth index used the principal component analysis method [33–35].All the variables which were used in the paper are defined in Table 1.

**Table 1:** Description of explanatory variables.

Age	Is a binary variable taking either 1 or zero for each of the ages 15 to 19
Early Sexual Debut	Is a binary variable taking the value of 1 if the adolescent had her first sexual intercourse below the age of 16 and, 0 if later or, had never had sex before the date of the interview
Knowledge of contraception method	Binary variable with 1 if the respondent knew any modern contraception method and 0 otherwise.
Socioeconomic status	Categorical variable defined in five quintiles of a wealth index; 1 = Wealth quintile 1, 2 = Wealth quintile 2, 3 = Wealth quintile 3, 4 = Wealth quintile 4, and 5 = Wealth quintile 5
Marital status	Binary variable capturing the marital status of the respondent; 1 = married, 0 otherwise.
Education	A categorical variable of the highest level of education that the adolescent has ever attained: 1 = no education, 2 = primary education, 3 = secondary education and 4 = any post-secondary education (such as college diploma, and university degree).
Place of residence	Binary variable with 1 if urban area and 0 otherwise.
Region	Categorical variable with 1 = southern region, 2 = central region, and 3 = northern region.
Sex of household head	Binary variable with 1 = male household head, and 0 = female household head
Media exposure	This was constructed from weighting the frequency of watching television, listening to radio and reading newspapers and magazines, We then constructed a categorical variable with 1 = no media exposure, 2 = irregular media exposure, and 3 = regular media exposure
Religious affiliation	Categorical variable with 1 = no religion, 2 = Christian, and 3 = Moslem

**Notes:** All categorical variables were entered as dummy variables taking a value of 1 or 0 otherwise

### Statistical analysis

The analytical approach was done in four stages. First, we calculated the summary statistics of all the variables used in the study to show the distribution of the sample. In the second stage we constructed concentration curves (CC) to explore the existence of inequalities in teenage pregnancy and childbearing. The CC provides a pictorial view of the pattern of inequality in teenage pregnancy and childbearing. The CC plot the cumulative percentage of teenage pregnancy and childbearing on the *y*-axis and wealth status ranked by cumulative percentage of the population on the *x*-axis. For our variable of interest, if the CC lies above the line of equality (45^o^ straight line from the origin), it means there is pro-poor inequality in teenage pregnancy and childbearing. Pro-rich inequality exists if the CC lies below the equality line. A perfect situation of no socioeconomic inequality in teenage pregnancy and childbearing prevails if the CC coincides with the line of equality.

Although it gives a good glimpse of inequality, the CC does not quantify the extent of inequality which exists in the variable under study. Therefore, in the third stage, we calculated concentration indices (CI) to estimate the degree of socioeconomic-related inequality in teenage pregnancy and childbearing–a procedure routinely used in the literature to measure socioeconomic-related inequality in health or health-related variables [36–38]. The CI measures “twice the area between the concentration curve and the line of equality” [39]. The method has been widely used to measure inequality in maternal healthcare utilisation and child health among others [40–42]. Finally, in the fourth stage we decomposed the CI to untangle the sources of socioeconomic-related inequality in teenage pregnancy and childbearing.

In this paper we used the Erreygers corrected concentration index (*EI*) in our assessment of the levels of socioeconomic inequality in teenage pregnancy and child bearing. The *EI* is expressed as;

$$EI=8*cov(h_{i},r_{i})$$ (1)

where *EI* is the covariance between individual health (*h*_*i*_) and the individual’s relative rank in wealth distribution (*r*_*i*_). The *EI* ranges between -1 and +1. A negative (positive) index mean that ill-health is concentrated in individuals with relatively low (high) income (wealth). If *EI* is zero, no income-related inequality in the distribution of ill-health exists. Assuming that teenage pregnancy and childbearing (*h*) is linearly related to its determinants, the *EI* above can then be written as a linear function of *K* determinants *x*_*k*_,[43] then the relationship can be expressed as;

$$h_{i}=\emptyset +\sum_{k=1}^{K}\beta_{k}x_{ik}+\epsilon_{i}$$ (2)

where *h*_*i*_ is the health outcome measure (teen pregnancy and childbearing), *β*_*k*_ is the partial effect of a regressor, and *x*_*ik*_ are explanatory variables. Thus, the *EI* can then be substituted for *h*_*i*_ and then decomposed into its determinants. Eq (2) is then estimated using an ordinary least square (OLS) regression model and decomposed as;

$$EI=4[\sum_{k=1}^{K}\beta_{k}{\bar{x}}_{k}C_{x_{k}}+{GC}_{\varepsilon }]$$ (3)

where ${\bar{x}}_{k}$ is the mean of *x*, $C_{x_{k}}$ is the CI for determinant *k*, and *GC*_*ε*_ is the generalised concentration index of the residual. All the data were analysed in Stata 15.

## Results

### Socioeconomic and demographic characteristics

Table 2 shows that the percentage of teenage pregnancy and childbearing in 2004 was 34.1% and this percentage declined to 25.6% in 2010 and then increased to 29% in 2015–16. The average percentage over the period was 28.7%. The results in the table also indicate that there was slight change in early sexual debut over time at each survey point—which was at 36.7%, 33.4% and 36.4% in 2004, 2010, and 2015–16, respectively. Knowledge of modern methods of contraception increased over time: about 72% (2004) and 76% (2015–16) of teenagers in the sample at least knew of one modern family planning method.

**Table 2:** Demographic and socioeconomic characteristics.

	2004 (N = 2,407)		2010 (N = 5,039)		2015–16 (N = 5,273)		Pooled (N = 12,719)	
Variables	%	n	%	n	%	n	%	n
Teen pregnancy and childbearing	34.1	821	25.6	1,290	29.0	1,529	28.7	3,650
Age 15	18.6	448	24.7	1,245	23.8	1,255	23.2	2,951
Age16	19.5	469	23.0	1,159	17.9	944	20.6	2,620
Age 17	17.8	428	18.5	932	18.4	970	18.1	2,302
Age 18	23.2	558	18.1	912	20.4	1,076	20.0	2,544
Age 19	20.9	503	15.7	791	19.6	1,034	18.1	2,302
Early sexual debut	36.7	883	33.3	1,678	36.4	1,919	35.8	4,553
Knows contraception method	72.0	1,733	70.1	3,532	76.4	4,029	73.5	9,348
Wealth quintile 1	26.3	633	25.1	1,265	23.4	1,234	24.1	3,065
Wealth quintile 2	21.4	515	19.7	993	19.3	1,018	21.1	2,684
Wealth quintile 3	18.6	448	19.7	993	19.9	1,049	19.2	2,442
Wealth quintile 4	17.2	414	17.8	897	19.1	1,007	17.9	2,277
Wealth quintile 5	16.5	397	17.8	897	18.3	965	17.6	2,239
Married	32.9	792	23.4	1,179	23.5	1,239	25.0	3,180
Higher education	0.4	10	0.7	35	0.4	21	0.3	38
Secondary	19.9	479	23.1	1,164	26.6	1,403	24.5	3,116
Primary	74.6	1,796	73.3	3,694	70.4	3712	72.3	9,196
No education	5.1	123	2.9	146	2.6	137	2.8	356
Urban	19.0	457	18.9	952	17.4	918	17.2	2,188
Southern	43.9	1,057	44.1	2,222	45.9	2,420	46.9	5,965
Central	40.6	977	43.5	2,192	42.9	2,262	34.7	4,413
Northern	15.5	373	12.3	620	11.2	591	18.4	2,340
Male household head	73.9	1,779	68.2	3,437	67.1	3,538	68.8	8,751
No Media exposure	15.9	383	16.7	842	45.9	2,420	28.1	3,574
Irregular media exposure	13.9	335	18.0	907	18.8	991	17.0	2,162
Regular media exposure	70.2	1,690	65.3	3,290	35.3	1,861	54.9	6,983
No religion	0.7	17	0.4	20	0.3	16	0.4	51
Christian	88.6	2,133	87.6	4,414	87.7	4,624	88.5	11,256
Moslem	10.7	258	12.1	610	12.1	638	11.1	1,412

Over the years a higher percentage of teenagers had primary education (about 72.3%) compared to those who had secondary or higher levels of education (about 25%). Almost 73.9% were from male-headed households in 2004, 68.2% in 2004 and 68.8% in 2015–16. Regular media exposure seemed to have declined considerably in 2015–16. However, this could be an indication of a change in taste in the forms of media used, which was not captured in the surveys.

### Teenage pregnancy and childbearing by wealth status

**Fig 1:**
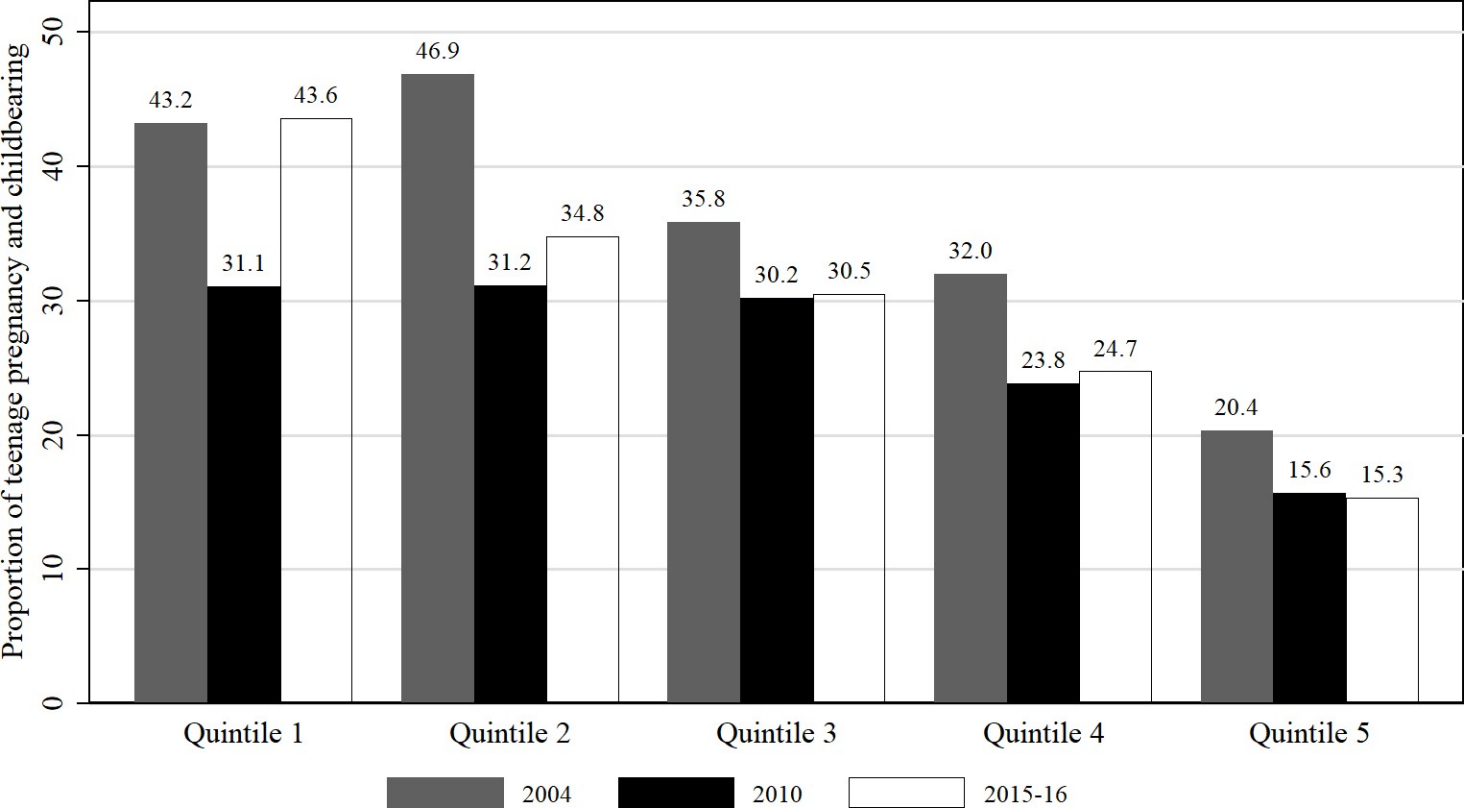
Teenage pregnancy and childbearing trend by wealth status 2004-2015-16. Constructed by authors from MDHS 2004, 2010, 2015–16.

From Fig 1, a downward trend in teenage pregnancy and childbearing by wealth status can be observed. For the poorest category, the lowest rate was in 2010, at around 31.1%. The percentage of teenage pregnancy and childbearing was still higher, and no substantial difference between 2004 and 2015–16. For the wealth quintile 3, the prevalence remained almost the same, between 2010 (30.2%) and 2016 (30.5%). Suffice to say, the respondents in wealth quintile 5 had the lowest level of teenage pregnancy and childbearing; 20.4% in 2004, 15.6% in 2010 and 15.3% in 2015–16. In general, the results show that teenage pregnancy and childbearing did not decline much among the group in wealth quintile 1, but the prevalence declined in all other categories between 2004 and 2015–16.

**Fig 2:**
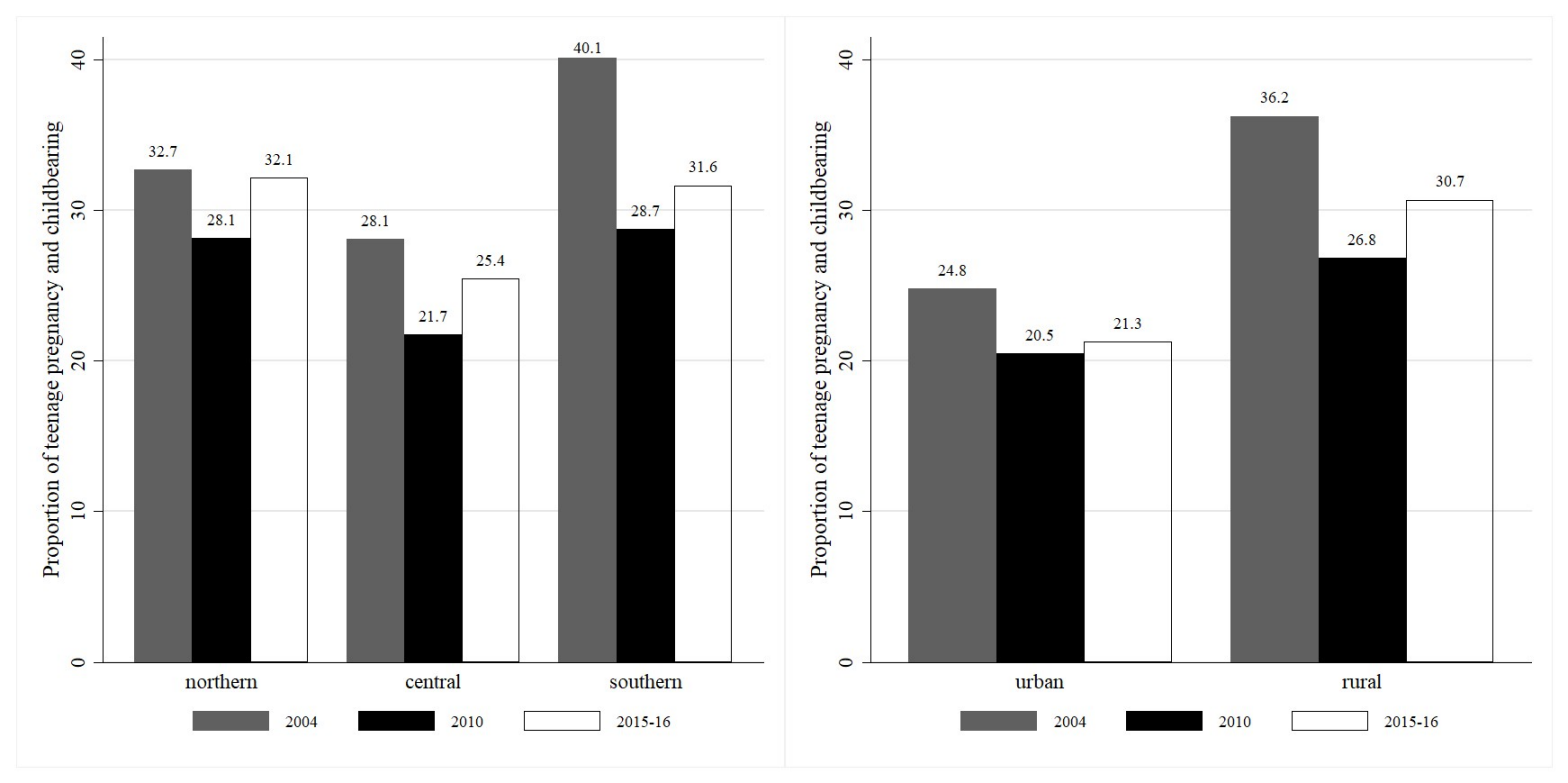
Teenage pregnancy and childbearing trend by region and residence 2004-2015-16. Constructed by authors from MDHS 2004, 2010, 2015–16.

With respect to regional variations in Fig 2, in all the years the prevalence was higher in the southern region (40.1% in 2004, 28.7% in 2010 and 31.6% in 2016–15). The northern region came in second with 32.7% in 2004, 28.1% in 2010 and 32.1% in 2015–16. The prevalence was lower in the central region (28.1% in 2004, 21.7% in 2010 and 25.4% in 2015–16). In terms of rural-urban differences, rural areas had a higher prevalence of teenage pregnancy and childbearing. However, the percentage decline in teenage pregnancy and childbearing over the years in rural areas was higher than in urban areas.

### Inequity in teenage pregnancy and childbearing

**Fig 3:**
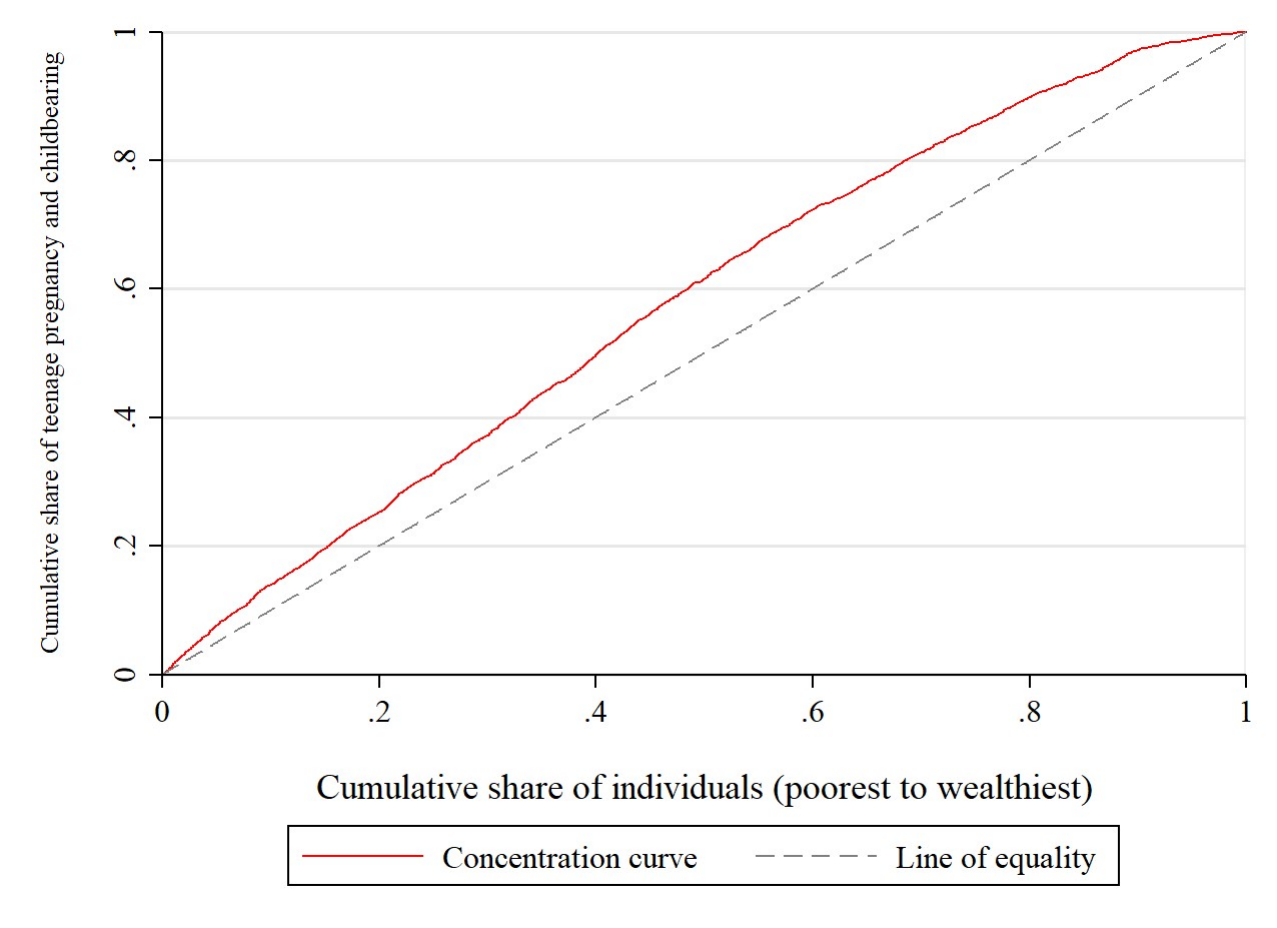
Concentration curve for teenage pregnancy and childbearing (Pooled data). Constructed by authors from MDHS 2004, 2010, 2015–16.

**Fig 4:**
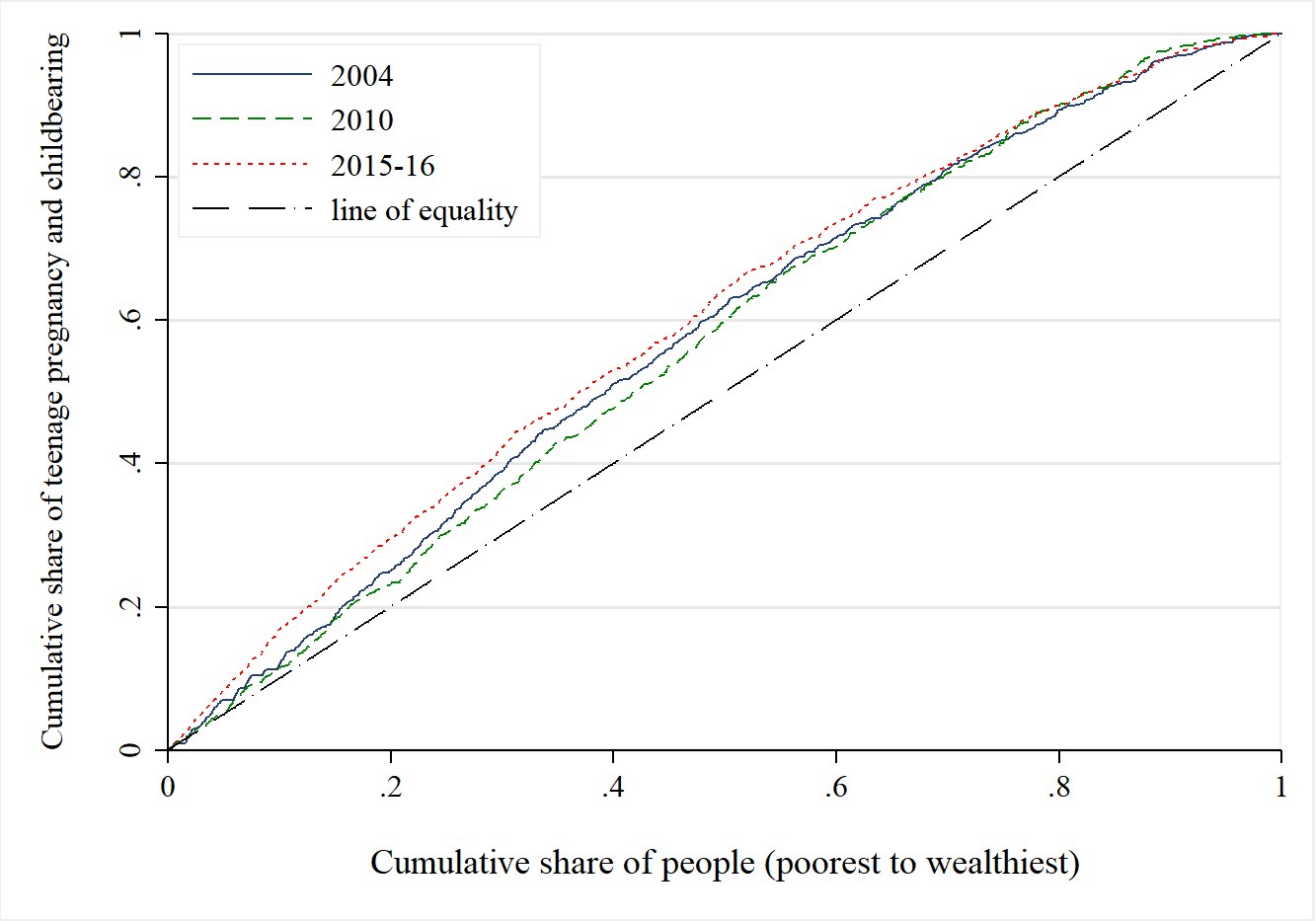
Concentration curve for teenage pregnancy and childbearing 2004–2016. Constructed by authors from MDHS 2004, 2010, 2015–16.

Using CCs, Figs 3 and 4 show that inequality in teenage pregnancy and childbearing favoured the poor. That is to say that teenage pregnancy and childbearing was concentrated among the poor. The CCs consistently diverged from the line of inequality with time, suggesting a worsening of socioeconomic inequality over the period. This worsening in inequality was much higher among teenagers who were from poorer households than those from richer households. The difference was concentrated up to about 54% of the distribution, as shown in all the curves, thereafter the difference becomes more negligible at the higher wealth quintiles.

There is no apparent difference in terms of magnitude among the curves. Due to this obscure picture, the need for a summary measure is essential. This calls for the concentration indices, and these are presented in Table 3.

**Table 3:** Concentration indices for teenage pregnancy and childbearing.

	2004	2010	2016–15	Pooled
Concentration index	-0.207***	-0.133***	-0.217***	-0.187***
	(0.021)	(0.014)	(0.014)	(0.009)
*N*	2407	5039	5273	12719

**Notes:** Standard errors in parentheses

* *p* < 0.10

** *p* < 0.05

*** *p* < 0.01

In Table 3, all concentration indices are negative and statistically different from zero indicating pro-poor socioeconomic inequality in teenage pregnancies and childbearing, thereby supporting the results from the CCs in Fig 4. Examining the trend, *EI* for teenage pregnancies and childbearing changed from -0.207 (*p*<0.01) in 2004 to -0.133 (*p*<0.01) in 2010, and finally to -0.210 (*p*<0.01) in 2016–15. While this pattern is mixed, over the entire period socioeconomic inequality worsened to the disadvantage of the teenage girls who were from poorer backgrounds. Given the evidence of the existence of socioeconomic inequality in teenage pregnancy and childbearing above, we decomposed the CI to determine the contributing factors and how these explain the observed differences. For brevity, we only present results showing the contributions of each of the determinants to the observed socioeconomic inequality in teenage pregnancies and child bearing.

**Fig 5:**
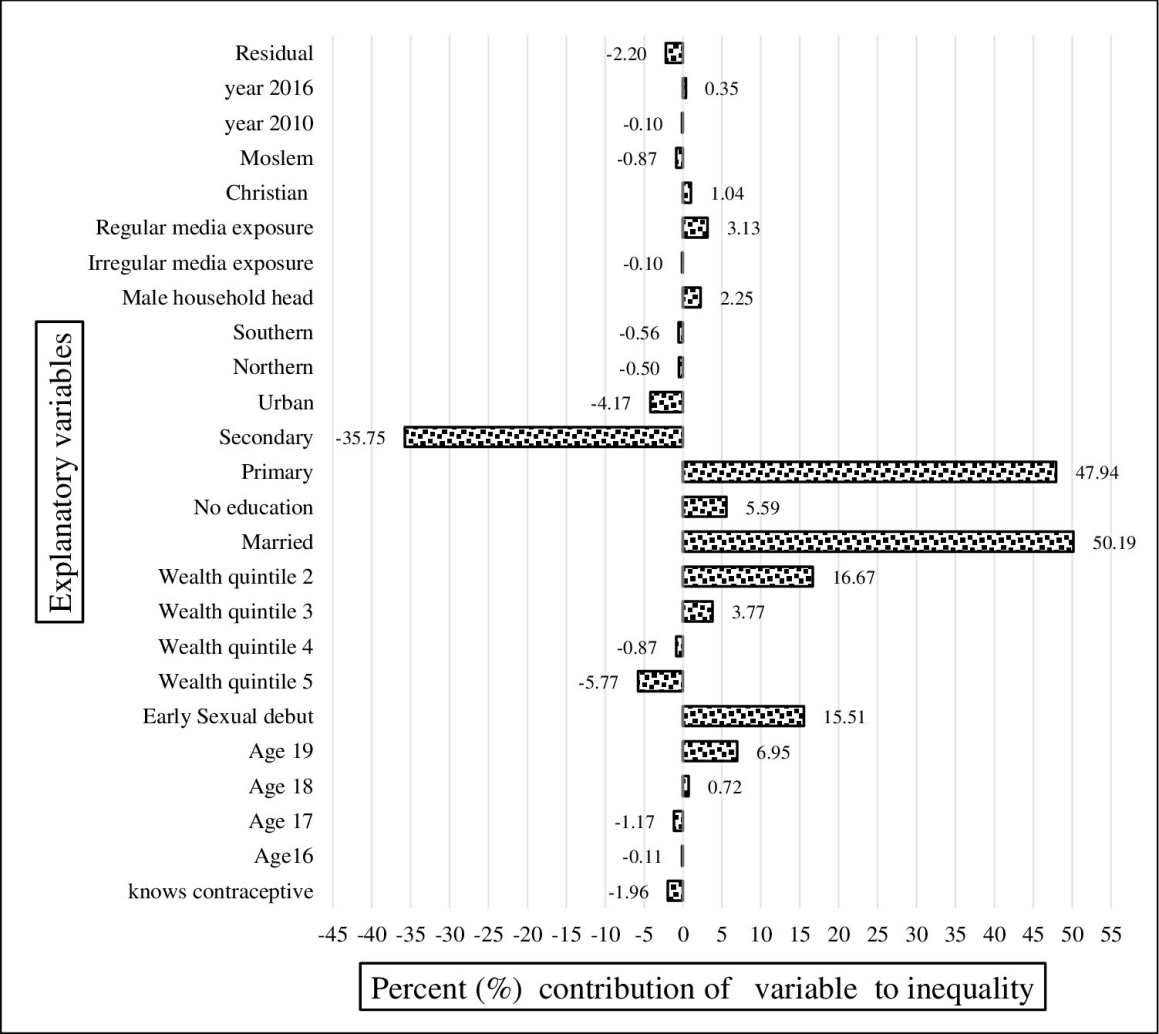
Decomposition of the concentration index for the pooled data. Constructed by authors from MDHS 2004, 2010, 2016.

In Fig 5 we show the decomposition results from the pooled sample. The *y*-axis measures the percentage contribution to the socioeconomic inequality in teenage pregnancy and child bearing of each of the variables in the regression model whereas the *x*-axis indicates the variable of interest. The results in the tables show that having an early sexual debut, being married, being 19-years-old, and having no or only primary education contributed a significant share to the observed inequality in teenage pregnancy and childbearing. Having secondary education reduced the overall inequality. Mass media exposure was also shown to be a significant contributor to the wealth-related inequality in teenage pregnancy and childbearing.

**Fig 6:**
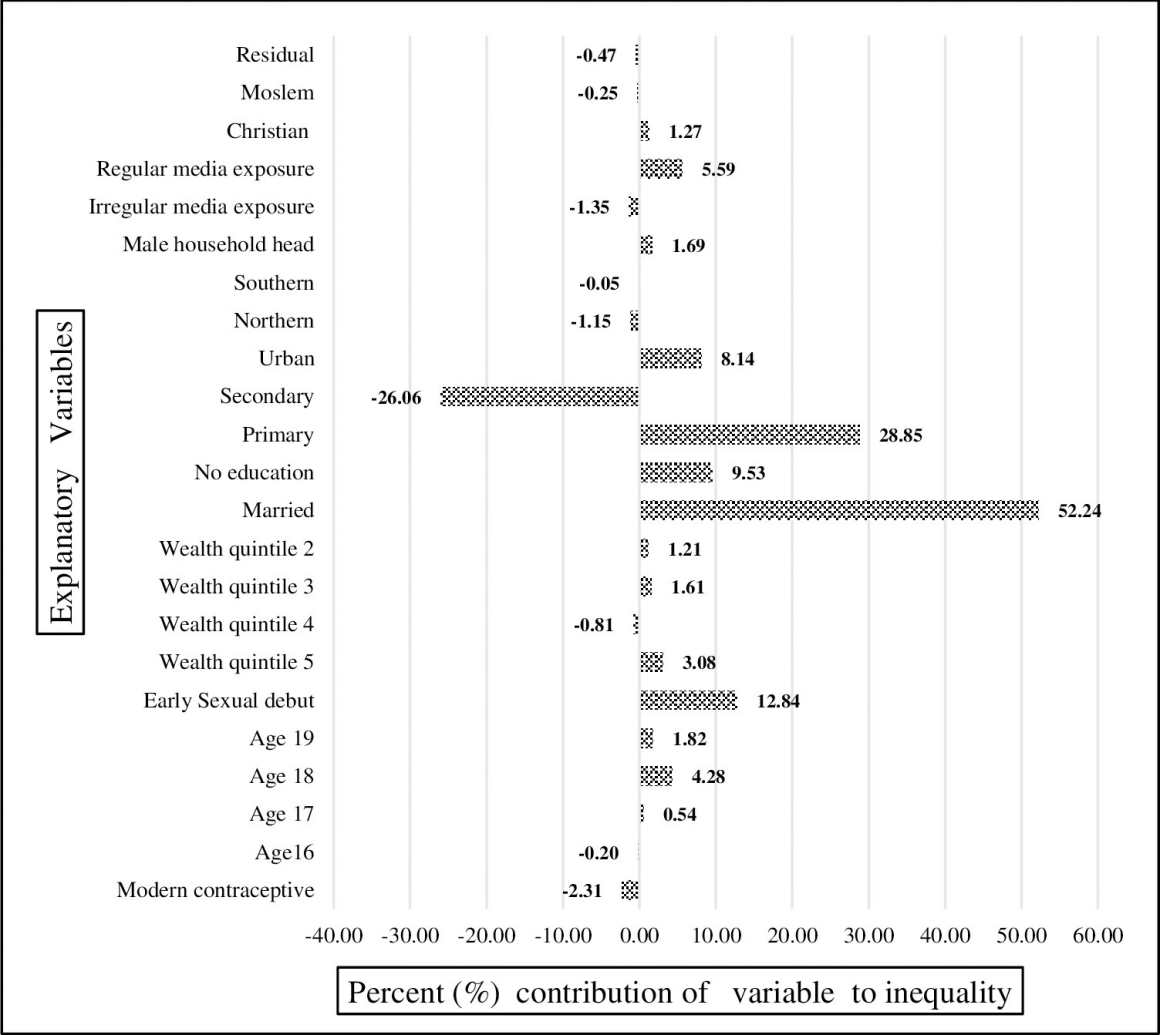
Decomposition of the concentration index for year 2004. Constructed by authors from MDHS 2004, 2010, 2015–16.

**Fig 7:**
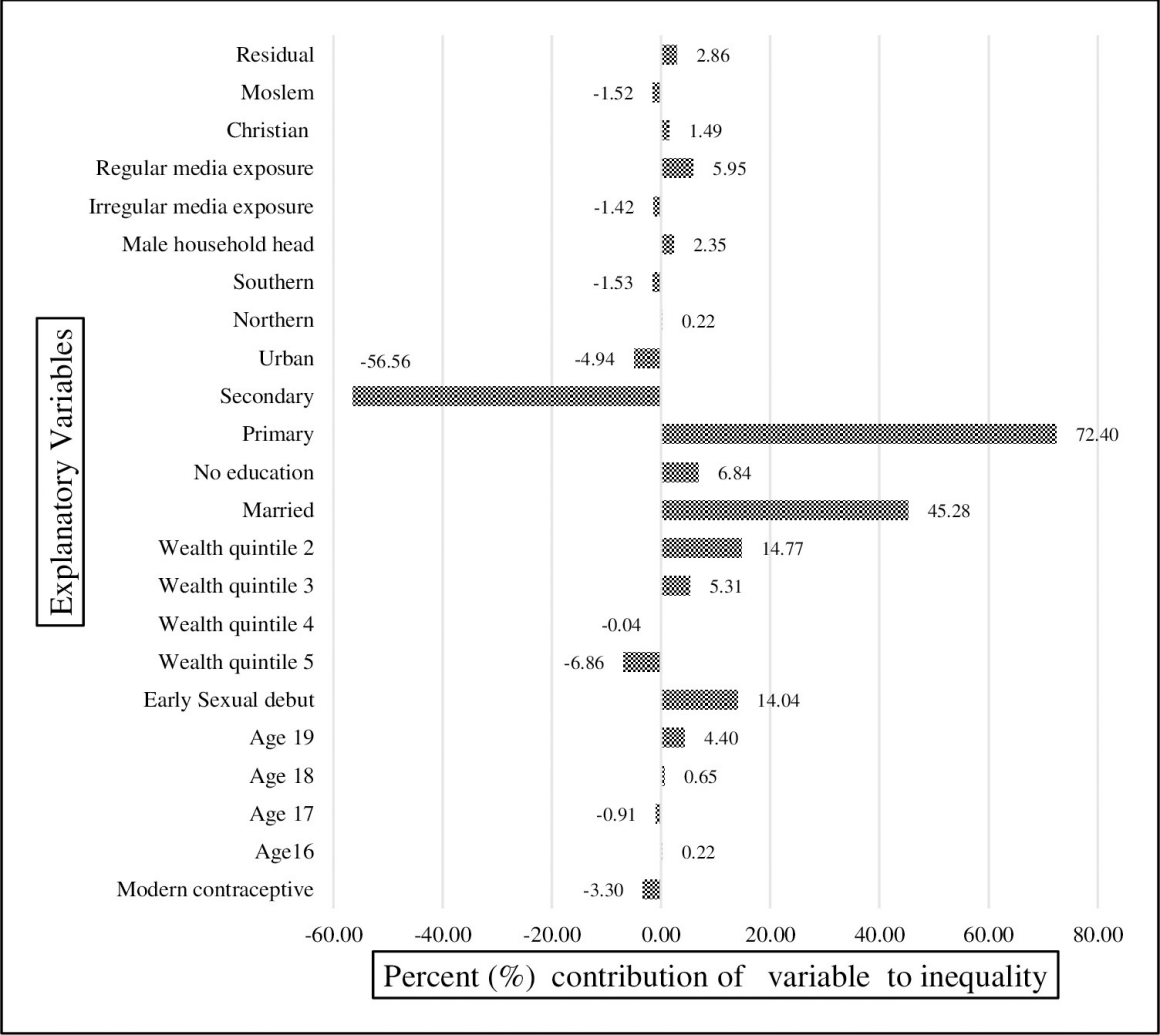
Decomposition of the concentration index for year 2010. Constructed by authors from MDHS 2004, 2010, 2015–16.

**Fig 8:**
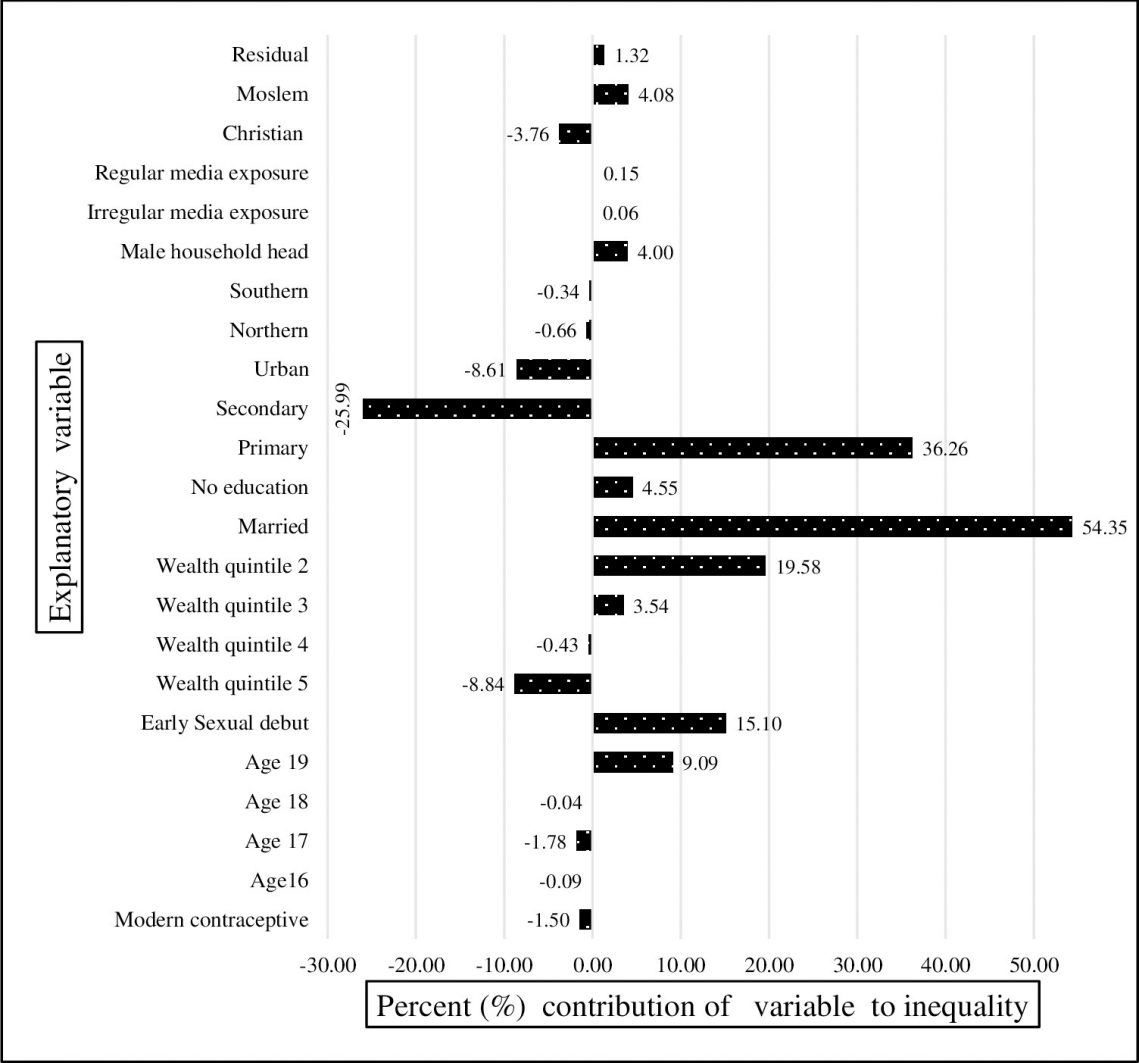
Decomposition of the concentration index for year 2016. Constructed by authors from MDHS 2004, 2010, 2015–16.

In Figs 6, 7 and 8, for each year, knowledge of modern contraception contributed negatively to wealth-related inequality in teenage pregnancy and childbearing. It accounted for almost -2.13% in 2004 (Fig 6), -3.3% in 2010 (Fig 7), and-1.5% in 2015–16 (Fig 8). Just as in the result from the decomposition of the pooled sample, early sexual debut contributed positively to wealth-related inequality in teenage pregnancy and childbearing, accounting for 12.8% in 2004 (Fig 6), 14.04% in 2010 (Fig 7), and 15.5% in 2015–16 (Fig 8). In terms of the direct effect of wealth, the results show that the aggregated effect was about 13.8% over the entire period (in the pooled decomposition), which suggests the increasing wealth-related inequality in teenage pregnancy and childbearing. However, a large share of inequality contributions emanated from inequality in education.

## Discussion

In this paper, we have measured the wealth-related inequalities in teenage pregnancy and childbearing using the EI. We have also assessed the trend in the overall inequality and then employed the decomposition method to establish the underlying factors explaining the observed inequalities in teenage pregnancy and childbearing over time. To the best of our knowledge, this is the first study for Malawi that estimates and decomposes socioeconomic inequality in teenage pregnancies and childbearing in order to understand the underlying factors and their contribution to the observed socioeconomic inequality.

Our results point to three critical messages. Firstly, teenage pregnancy and childbearing declined, but by very little, within the reference period. We found evidence of pro-poor socioeconomic inequality in teenage pregnancy and childbearing in Malawi. Secondly, upon examining the sources of the socioeconomic inequality, the decomposition analysis of the wealth-related inequalities in teenage pregnancy and childbearing demonstrates that inequality in education and early sexual debut were among the biggest contributing factors to teenage pregnancy and childbearing. Thirdly, we found that being married contributed about 50% of the inequality in teenage pregnancy and childbearing, followed by early sexual debut which accounted for 15.5% of the wealth-related inequality in teenage pregnancy and childbearing.

With respect to wealth status, the direct contribution of inequality in wealth accounts for approximately 13.8% in inequality in teenage pregnancy and childbearing. Since there is no comparable study that undertakes decomposition of this type, it is hard to directly compare the result of the concentration indices and decomposition analysis. However, our results may fall in line with previous studies that established that early teenage sexual debut, education, and wealth are among the important determinants of teenage pregnancy in Malawi [21,44,45] as well as in other countries [22,24,25]. The closest study to our paper, decomposed factors associated with unintended pregnancy in Iran [28]. That study found that inequality in the distribution of wealth contributed more to the inequality in unintended pregnancy and accounted for almost 27% of the variation.

Apart from the above, we also found that knowledge of modern contraception was concentrated among the rich in all the survey years. Since the overall concentration index was negative, the concentration index for knowledge of contraceptive methods was positive, whereas its relative contribution was negative. It means that wealth-related inequality in teenage pregnancy and childbearing was explained by wealth-related inequality in knowledge of contraceptive methods and their determinants. In terms of early sexual debut, the negative concentration index values for all the years suggest that sexual debut was also concentrated among the poor. After controlling for year fixed effects, in 2010, the teenage pregnancy and childbearing was concentrated among the richer, whereas in 2016, it was concentrated among the poor.

The effect of media exposure on inequality in teenage pregnancy and childbearing was mixed for the reference period. We found that irregular media exposure was concentrated among the poor, whereas regular media exposure was concentrated among the rich. Since the overall absolute contributions were all positive whereas the overall concentration index for teenage pregnancy and childbearing was negative, this suggests that the relative contribution was negative. Hence, mass media exposure reduces wealth-related inequality in teenage pregnancy and childbearing.

There are possible reasons that can help to understand the results which we have obtained. Firstly, there was worsening inequality in wealth and income growths to the disadvantage of the poor over the period in the country [39]. This may have potentially led the girls from low-income families to engage in early sexual debut and marriages as a way of coping. Hence, having the inequality in the distribution of wealth exacerbated the inequality in teenage pregnancies and child bearing. Secondly, most of the programmes that have been implemented as a way to prevent girls from low-income families falling pregnant in the early stages of their lives, have been run by Non-Governmental Organisations, and they have lacked continuity beyond their project phase. This may probably explain the marginal decline in teenage pregnancy and childbearing. Lastly, in recent times, the influence of some local leaders in dissolving teenage marriages and imposing penalties on the guardians if one of their teenage girls becomes involved in such unions, may have resulted in reducing teenage pregnancies. For example, in Dedza district in the country, almost 850 underage teenage marriages were dissolved [46].

Our paper has its strengths and shortcomings. The main strength of the paper is that using nationally representative data for the first time, we have shown the key elements which explain the inequality in teenage pregnancy and childbearing in Malawi. Furthermore, by using the decomposition method, we have accounted for the main factors underlying the disparities in teenage pregnancy and childbearing. However, the limitation lies in the fact that decomposition methodology is only an accounting exercise, hence the results cannot necessarily be interpreted as causal because we do not take endogeneity into account. Furthermore, we did not consider the role of supply-side factors in explaining the observed inequalities in teenage pregnancy and childbearing. This is a result of the fact that the data could not sufficiently provide the variables. Despite the limitations, the results have some important implications for future research. As a way forward, future researchers should also consider using other methods such as a propensity score and instrumental variables to control for endogeneity. Furthermore, it may also be interesting to employ the new methods which allow for decomposition beyond the mean [47].

## Conclusion

Inequality in teenage pregnancy and childbearing exists in Malawi and its prevalence has been worsening to the disadvantage of the poor. The observed pro-rich distribution in disparities in teenage pregnancy and childbearing is mainly accounted for by early sexual debut, education, marriage, and household wealth. In terms of policy, it means that there is a need for further strategies in Malawi to target girls from low-income families with more educational support programmes and civic education relating to early marriages and sexual relationships. Some of the programmes may be conditional cash transfers to keep vulnerable girls in school. Furthermore, civic education through social media platforms may be of more help to the teenagers. This could be a worthwhile approach because the teenage group is more associated with the use of social media.
